# Pre-Service Teachers’ Perceptions of and Experiences with Classroom Physical Activity

**DOI:** 10.3390/ijerph20021049

**Published:** 2023-01-06

**Authors:** Hannah Bigelow, Barbara Fenesi

**Affiliations:** Faculty of Education, Western University, London, ON N6G 1G7, Canada

**Keywords:** pre-service teachers, daily physical activity, classroom physical activity

## Abstract

Physical inactivity is one of the most modifiable factors linked to childhood obesity. Several Canadian provinces adopted daily physical activity (DPA) policies to promote physical activity during the school day. In Ontario, only 23% of in-service teachers meet DPA mandates. Promoting DPA implementation must occur at the pre-service level to foster self-efficacy and create long-term teaching habits. This study surveyed 155 pre-service teachers from an Ontario university to determine key perceptions and practices that should be targeted during their educational training to improve DPA fidelity. Findings revealed that over 96% of pre-service teachers viewed physical activity as beneficial for their own and their students mental and physical health, and as much as 33% received no education or training related to DPA. Pre-service teachers valued DPA more if they had opportunities to learn about and observe DPA during school placements. Pre-service teachers were more confident implementing DPA if they were more physically active, viewed themselves as more athletic, and had more positive physical education experiences. This work brings to the forefront important factors that could contribute to DPA implementation among in-service teachers and highlights target areas at the pre-service level for improved fidelity.

## 1. Introduction

Childhood overweightness and obesity is one of the most serious global health crises [1]. Among Canadian children, 30% are classified as overweight or obese, triple that of 30 years ago [2]. If this trend continues, it is projected that more than 50% of Canadian adults will be overweight or obese by 2030, presenting a substantial economic and health strain on society [2,3]. Childhood obesity can have long-term health complications that follow into adulthood, such as heart disease, cancer, and diabetes, but it can also have acute effects on sleep quality, hormone and respiratory function, and musculoskeletal development [4,5,6,7,8]. Childhood obesity also increases the risk of poor social and emotional health [9] often contributing to poor self-image, self-isolation, and clinical depression [8,10,11,12]. Furthermore, overweight children are four times more likely to report having academic challenges compared to their normal weight peers [4,5,6,7,8,9,10,13].

Several factors contribute to weight-related health concerns among children, including socioeconomic status, poor nutrition, environmental issues, and genetic predispositions [14,15]. While many factors are challenging to remedy, physical inactivity and sedentariness are two of the most modifiable and impactful factors linked to obesity [4,14,15]. It is estimated that a 25% reduction in the rate of physical inactivity would prevent an estimated 1.3 million deaths a year globally [16]. Increasing opportunities for children to be more physically active is a critical step towards addressing the obesity crisis among Canadian youth.

Physical activity is defined as any bodily movement generated by skeletal muscles that requires energy expenditure above resting metabolic rate; this includes play, work, house chores, recreational activities, and active transportation such as biking [17,18]. The benefits of physical activity for children are seen across several physical and cognitive domains, including but not limited to general physiology, increased strength and endurance, neurogenesis, reduced risk of disease, neuroimmune functioning, self-esteem and psychological well-being, emotional regulation, cognitive functioning and academic achievement [19,20,21,22,23,24,25,26,27]. It is recommended that children and youth aged 5–17 engage in physical activity for at least 60 min per day of moderate to vigorous intensity [28]. However, approximately 60% of Canadian children and youth are not meeting this guideline [29]. In addition, physical inactivity has been exacerbated by the COVID-19 pandemic, with a further 20% reduction in daily physical activity among children and youth [30].

Given that children spend most of their day in school, elementary classrooms serve as an ideal setting to increase physical activity opportunities [31]. Children who participate in classroom-based physical activity are more likely to meet the World Health Organization’s (WHO) recommendation of 60 min of daily physical activity [32] are better able to concentrate and remain on-task during lessons [33,34,35], have fewer disruptive behaviours such as fidgeting [35,36], have higher motivation and engagement in school related learning [37], have higher mood and self-efficacy [38,39] and have higher overall academic achievement than children in traditional sedentary classrooms [21,40,41,42]. Some of these results (i.e., improved on-task behavior and academic achievement) can be seen with as little as four minutes of in-class physical activity [43]. Classroom-based physical activity also supports school social climate, promoting overall connectedness between students, peers, and teachers, as well as increasing feelings of comfort and safety in the classroom environment [44,45].

To promote classroom-based physical activity, several Canadian provinces have adopted daily physical activity policies, including British Columbia, Alberta, Saskatchewan, Manitoba, and Ontario, representing approximately 72% of Canadian children aged 5–19 years [46]. Of particular interest to this study, the Ontario Ministry of Education released the Daily Physical Activity (DPA) policy in elementary schools in 2005. This policy requires publicly funded school boards to ensure that all students, including those with disabilities, grades 1 through 8, have a minimum of twenty minutes of moderate to vigorous physical activity each school day during instructional time [47]. However, a recent evaluation found that only 23% of Ontario teachers were meeting the DPA mandate [48]. Similarly, accelerometry data across 16 schools in Ontario also showed that fewer than half of children were provided with adequate DPA [49].

The social ecological model [50,51] and the social cognitive model [52,53] have been previously used to uncover key barriers of DPA implementation. The social ecological model suggests that behaviour reflects complex dynamic interrelations among personal and environmental factors, whereas the social cognitive model suggests that behavioral self-efficacy develops by being afforded opportunities to observe and interact with others who are knowledgeable within a certain domain. Taken together, these models highlight that barriers to and facilitators of DPA can be found within and between teachers, among students, principals, school boards, community values and institutional policy. Several barriers to DPA implementation have been identified, with insufficient training and education consistently noted as two of the most salient barriers [48,54,55,56,57]. Teachers have noted that their education degrees did not include information about the benefits of classroom-based physical activity or how to implement activities in their future classrooms; this lack of education and training contributed to reduced motivation and confidence implementing physical activity in their classrooms [57,58,59]. Another important barrier to DPA implementation is teachers’ perceptions of the value of physical activity for their own and their students’ physical and mental health. Specifically, teachers who view physical activity more positively and who are more physically active themselves, are more likely to implement classroom-based physical activity [60,61,62]. In addition, teachers who view themselves as athletic or sporty [63,64], or who have had positive prior experiences with sport or physical activity [64,65,66] are more likely to implement physical activity in their classrooms.

It is evident that educational training, personal beliefs, and prior experience have a significant impact on teachers’ willingness to implement DPA. Unfortunately, there is substantial evidence to suggest that teaching beliefs and practices become engrained over time and grow increasingly resistant to change [67,68,69,70]. While there are professional development opportunities for in-service teachers to update their classroom practices, with many targeting DPA implementation [55,71,72], the honing of these skills must also occur at the pre-service level to promote self-efficacy and create sustainable teaching habits. However, there has been minimal work examining pre-service teachers’ perceptions of and experiences with DPA. This work is vital to elucidate how to better support teachers’ DPA implementation skills prior to entering their teaching careers. Work in this area has mainly targeted pre-service teachers in physical education specialties and has found that those who had positive experiences with their own physical education classes [73] or who identified as sporty or athletic [74,75] had more confidence in their ability to teach physical education. To our knowledge, only one study has examined generalist pre-service teachers’ perceptions of DPA and found that lack of training and hands-on experience were notable barriers [76]. Prior physical activity experiences and personal physical activity behaviors also influenced pre-service teachers’ confidence with DPA [76]. More work is needed to identify the gaps in training, knowledge, and perceptions surrounding DPA among generalist pre-service elementary teachers, as they hold significant responsibility in getting children active. Focusing on these gaps at the pre-service rather than the in-service level will help identify proactive ways to support DPA fidelity.

The current study surveyed pre-service teachers at the beginning and end of their pre-service education program to target the aforementioned gaps in our understanding of DPA fidelity by addressing three research goals: Goal 1: (a) To characterize pre-service teachers’ perceptions of physical activity as it relates to their own health and to their students’ health, (b) to characterize pre-service teachers’ perceptions of the role of physical activity in the school and classroom, and (c) to characterize pre-service teachers’ experience with DPA implementation during their educational training. Goal 2: To determine key factors related to pre-service teachers’ perceptions of (a) DPA value, and (b) confidence implementing DPA. The key factors examined were pre-service teachers’ perceptions of physical activity, their physical activity behavior, their physical activity identity, their previous physical education experience, and their experience during school placements (practicum). Goal 3: To evaluate whether there were changes in pre-service teachers’ (a) perceptions of DPA value and (b) confidence implementing DPA from the beginning to the end of their education program.

The ultimate goal of this work is to determine key characteristics that educators of pre-service teachers should target when training them on DPA implementation in order to improve fidelity.

## 2. Materials and Methods

### 2.1. Survey Design and Respondents

Respondents were recruited during their first year of a two-year Bachelor of Education program at an Ontario university from 11 September to 31 October 2020. At the beginning of the program (Year 1), we recruited 125 pre-service teachers using an electronic poster posted to their learning management system. Fifteen respondents did not complete all components of the survey or did not meet the inclusion criteria and therefore were removed from the dataset (*N* = 110). This represents a 34% response rate (*N* = 323 in the program). The survey was open to all respondents enrolled in the first-year program who intended to teach grades 1–8.

At the end of the program (Year 2), 78 pre-service teachers from the same program participated in this study. One respondent did not complete all components of the survey and therefore was removed from the dataset (*N* = 77); this represents a 31% response rate (*N* = 246 in program). The decline in program *N* reflects students who did not continue the program after their first year. The same inclusion criteria and recruitment method was used in Year 2 as was used in Year 1. Thirty-two respondents participated in both Year 1 and Year 2 of the study and represent the longitudinal data. Altogether, a total of 155 unique respondents participated in the study.

The Year 1 survey consisted of 42 questions and used a mix of multiple-choice, single choice, and short answer questions to query responders about their demographic information, their perceptions of DPA, attitudes toward physical activity in the classroom, and their own personal experience with physical activity. Respondents received CAD 10 for their participation in the form of a Starbucks gift card. The Year 2 survey consisted of 47 questions and used a mix of multiple-choice, single choice, and short answer questions to query respondents about their demographic information, their perceptions of DPA, their experience with and willingness to implement physical activity within their classroom, attitudes toward physical activity in the classroom, their training and education experience during their program, and their own personal experience with physical activity. Respondents received CAD 10 for their participation in the form of a Starbucks gift card. The study was fully approved by the institutions research ethics board.

### 2.2. Survey Questionnaires

School and Physical Activity Promotion Attitudes Questionnaire [77].

Several items from this questionnaire [77] were used to assess pre-service teachers’ perceptions of physical activity as it relates to their own and their students’ mental and physical health, as well as to assess pre-service teachers’ perceptions of the role of the school and classroom in physical activity promotion. To assess pre-service teachers’ perceptions of the value of classroom DPA, three questions were created using a 4-point Likert scale (1 = strongly disagree…4 = strongly agree) including: Classroom DPA improves the physical health of students; Classroom DPA improves cognitive functioning among students (e.g., their ability to pay attention, and complete tasks); I believe that students would demonstrate improved academic performance after participating in a bout of classroom physical activity.

#### 2.2.1. Teaching Physical Education Scale

Three items from this scale [78] were used to assess pre-service teachers’ identification with an athletic identity. Respondents were asked to rate their agreement on a 7-point Likert scale (1 = strongly disagree…7 = strongly agree) with statements: I think of myself as athletic; I think of myself as sporty; I think of myself as someone who is physically fit.

#### 2.2.2. Biographical Questionnaire

Three items from this questionnaire [77] were used to assess pre-service teachers’ prior experiences with physical education and activity in elementary and high school. Respondents rated their agreement with three items using a Likert scale from 1 to 4 (1 = strongly disagree…4 = strongly agree) including: I was good at physical education throughout school; My elementary physical education experiences were positive; My high school physical education experiences were positive. One additional item from the Biographical Questionnaire was used to assess confidence implementing DPA in future classrooms. Respondents rated their agreement with the statement I feel confident about implementing physical activity in my future classroom.

#### 2.2.3. International Physical Activity Questionnaire (IPAQ)

The IPAQ was used to assess pre-service teachers’ participation in moderate to vigorous physical activity per week (reported in minutes per week).

#### 2.2.4. Experience with DPA during Education Program

Several items were created to assess pre-service teachers’ experience with DPA during their education program. All pre-service teachers spend 20 weeks in schools practicing teaching to gain direct experience. This portion of the program is referred to as a practicum. According to Ontario DPA policy, all elementary school teachers should be implementing a minimum of 20 min per day of physical activity outside of regular physical education class or recess. Thus, pre-service teachers should have at the very least some exposure to DPA implementation through observation. Respondents were asked to respond to the following items: I had opportunities to observe DPA during my practicum (1 = strongly disagree…4 = strongly agree); Students were engaged in DPA (1 = strongly disagree…4 = strongly agree); Students enjoyed DPA (1 = strongly disagree…4 = strongly agree). Respondents were also asked more generally about whether their education training provided opportunities to learn about and prepare for DPA implementation, such as I had opportunities to learn about DPA during my pre-service education (1 = strongly disagree…4 = strongly agree); My pre-service education has prepared me for teaching DPA (1 = strongly disagree…4 = strongly agree).

### 2.3. Statistical Analyses

To address the first research goal, descriptive statistics (frequencies) for both surveys were used to characterize pre-service teachers’ perceptions of physical activity as it relates to their own and to their student’s health, to characterize pre-service teachers’ perceptions of the role of physical activity in the school and classroom, and to characterize pre-service teachers’ experience with DPA implementation during their educational training.

To address part (a) of the second research goal, Spearman’s correlations were used to assess the relation between pre-service teachers’ perceptions of the value of DPA and: perceptions of physical activity, personal physical activity behavior, personal physical activity identity, previous physical education experience, and practicum experience. Year 1 survey data were used for all analyses except for evaluating the relation between DPA value and practicum experience, as the practicum occurred in the second year of respondents’ education program. The variable representing “DPA value” was derived using a composite score from three items on the School and Physical Activity Promotion Attitudes Questionnaire (see Survey Items above). All three items were strongly correlated (rs > 0.344, ps < 0.001) and thus were combined into a composite score to reflect pre-service teachers’ perceptions of DPA value.

To assess the relation between pre-service teachers’ perceptions of DPA value and perceptions of physical activity, the composite DPA value variable was correlated with items from Table 1. To assess the relation between pre-service teachers’ perceptions of DPA value and their own physical activity behavior, the composite DPA value variable was correlated with pre-service teachers reported moderate to vigorous physical activity behavior in minutes from the IPAQ. To assess the relation between pre-service teachers’ perceptions of DPA value and their physical activity identity, a composite for physical activity identity was created using three items from the Teaching Physical Education Scale (see above); all items were strongly correlated (rs > 0.642, ps < 0.001). The composite DPA value variable was then correlated with the composite athletic identity variable. To assess the relation between pre-service teachers’ perceptions of DPA value and their previous physical education experience, a composite for previous experience was created using three items from the Biographical Questionnaire (see above); all items were strongly correlated (rs > 0.61 , ps < 0.001). The composite DPA value variable was then correlated with the composite physical education experience variable. To assess the relation between pre-service teachers’ perceptions of DPA value and their practicum experience, the DPA value composite from the Year 2 survey was correlated with items from Table 3.

To address part (b) of the second research goal, Spearman’s correlations were used to assess the relation between pre-service teachers’ confidence implementing DPA and: perceptions of physical activity, personal physical activity behavior, personal physical activity identity, previous physical education experience, and practicum experience. Year 1 survey data were used for all analyses except for evaluating the relation between confidence implementing DPA and practicum experience, as the practicum occurred in the second year of respondents’ education program. The item “I feel confident about implementing physical activity in my future classroom” from the Biographical Questionnaire was used to represent “confidence implementing DPA”. This item was correlated with the same items described above representing perceptions of physical activity, personal physical activity behavior, physical activity identity, previous physical education experience, practicum experience, and perceived value of DPA.

To address the third research goal, paired t-tests were used to evaluate whether there were changes in pre-service teachers’ (a) perceptions of DPA value and (b) confidence implementing DPA from the beginning to the end of their education program. Only respondents who completed both Year 1 and Year 2 surveys were included in analyses (*N* = 32). The same composite item was used to represent perceived DPA value; to represent confidence implementing DPA, the same item from the Biographical Questionnaire (I feel confident about implementing physical activity in my future classroom) was used.

To assess for survey validity, questions went through a Spearman Rank Order Correlation to measure the degree of association between questions belonging to the same questionnaire. All items were significantly correlated with the group total (all rs > 0.396, all ps < 0.01), indicating high validity.

## 3. Results

### 3.1. Goal 1

To characterize pre-service teachers’ perceptions of physical activity as it relates to their own health and to their students’ health (Table 1), (b) to characterize pre-service teachers’ perceptions of the role of physical activity in the school and classroom (Table 2) and (c) to characterize pre-service teachers’ experience with DPA implementation during their educational training (Table 3).

Regarding pre-service teachers’ perceptions of the benefits of physical activity, over 96% of pre-service teachers agreed that physical activity is beneficial for their own and their students’ mental and physical health. Over 93% of pre-service teachers also viewed being physically active as important to their identity as a teacher. However, only between 63 and 81% of pre-service teachers viewed being physically fit as important to their identity as a teacher.

Regarding pre-service teachers’ perceptions of physical activity in the school and classroom, over 97% of pre-service teachers viewed DPA as helpful to improve students’ physical health, cognitive functioning, and academic performance. Between 21 and 24% of pre-service teachers viewed DPA as detrimental to the cognitive functioning of students, but 6–8% believed increasing physical activity during the school day would be detrimental to students’ academic success. Furthermore, over 92% of pre-service teachers agreed that teachers should play a role in offering physical activity opportunities to students either in the classroom or more broadly within school programs, and that teachers can make a difference helping children adopt lifelong physical activity habits.

Regarding pre-service teachers’ experiences with DPA implementation during their educational training, only 66% noted having opportuntieis to observe DPA during their practicum. Of those who were able to observe DPA during practicum however, over 95% agreed that students engaged in and enjoyed DPA. Seventy-three percent noted having opportuniteis to learn about DPA during their pre-service education, and 70% agreed that their pre-service educaiton prepared them to teach DPA in their future careers.

### 3.2. Goal 2

To determine key factors related to pre-service teachers’ perceptions of (a) DPA value, and (b) confidence implementing DPA. The key factors examined were pre-service teachers’ perceptions of physical activity, their physical activity behavior, their physical activity identity, their previous physical education experience, and their experience during practicum. Results are shown in Table 4.

### 3.3. Goal 3

To evaluate whether there were changes in pre-service teachers’ (a) perceptions of DPA value and (b) confidence implementing DPA from the beginning to the end of their education program.

There was no change in teachers’ perceptions of DPA value from the beginning (*N* = 31; M = 3.81, SD = 0.36) to the end of their education program (*N* = 31; M = 3.71, SD = 0.44) t (30) = 1.10, *p* = 0.280). There was a significant decline in teachers’ confidence implementing DPA from the beginning (*N* = 29; M = 3.48, SD = 0.51) to the end of their education program (*N* = 29; M = 2.97, SD = 0.09) t(28) = 4.4, *p* < 0.001, d = 0.82).

## 4. Discussion

The current study aimed to characterize pre-service teachers’ perceptions of physical activity, to determine key factors related to their perceptions of DPA value and confidence implementing DPA, and to evaluate whether pre-service training leads to changes in perception of DPA and confidence in implementation. The following will discuss each goal in the context of previous findings and novel contributions and will offer suggestions for future research.

The overwhelming majority (96%) of pre-service teachers viewed physical activity as beneficial for their own and their students mental and physical health. Although most also viewed being physically active as important to their identity as a teacher (93%), being physically fit was viewed as less important (63–81%). This finding reflects a healthy dichotomy between the concept of a ‘physically active’ individual versus a ‘physically fit’ individual. Being physically active reflects a behavioral habit with tremendous heterogeneity in practice, whereas being physically fit reflects a personal accomplishment within the domain of physical health. Many have an ideal of physical fitness that is often outside their current experience, whereas engaging in physical activity is a choice that is almost always available. This is promising given that teachers who identify as physically active are more likely to recognize the benefits of physical activity for their students and implement it in their classrooms [60,61]. Further, students’ attitudes toward physical activity do not decline because of their teachers’ physical fitness levels [79,80]. Thus, physically active teachers of all fitness levels can serve as positive role models for their students, creating an inclusive and positive environment.

Interestingly, although nearly all pre-service teachers viewed DPA as beneficial to the student experience overall (over 97%), between 21 and 24% viewed DPA as detrimental to students’ cognitive functioning. However, this may not reflect a true belief that DPA is harmful to cognitive ability, but rather that engaging in DPA detracts from time that could be spent learning academic content. This notion is counterproductive as extensive evidence suggests that children who engage in classroom physical activity have higher academic achievement compared to children in traditional sedentary classrooms [21,40,41,42]. Given that only 6% of pre-service teachers reported that increasing physical activity during the school day would be detrimental to students’ academic success, it may be that some pre-service teachers simply view physical activity as belonging outside the classroom, but that increasing physical activity in general is beneficial. This is supported by the finding that over 92% of pre-service teachers agreed that teachers should play a role in offering physical activity opportunities to students either in the classroom or more broadly within school programs, and that teachers can make a difference helping children adopt lifelong physical activity habits.

When it came to pre-service teachers’ experience with DPA during their educational training, although 73% indicated that they had the opportunity to learn about DPA, 34% indicated that they did not receive any DPA exposure in their pre-service school placements. This finding aligns with prior work, with pre-service teachers similarly emphasizing a lack of hands-on experience during school placements [81]. Although most pre-service teachers learned about DPA during their education, many were unable to directly observe or practice DPA. Being able to observe an expert modelling to-be learned behavior is a critical step towards achieving self-efficacy and promoting future behavior [52]. Unfortunately, only half of in-service teachers are currently implementing DPA in Ontario [48,56], leaving pre-service teachers without sufficient role models for hands-on experience. Strategies toward better connecting teachers with available resources and improving access to information may be imperative in bridging this gap. This can simultaneously better educate in-service teachers about DPA and improve the experience of pre-service teachers during their school placements. Interestingly, despite these findings, most pre-service teachers (70%) reported that they were prepared to implement DPA in their future classrooms. Given the lack of hands-on experience, it is possible that pre-service teachers are over-inflating their confidence and ability to translate knowledge into practice, underscoring the need for further training at both the pre-service and in-service level. Critically, among those pre-service teachers who did have experience with DPA during school placements, over 95% reported that students enjoyed DPA. This is essential as positive experiences with DPA are likely to impact pre-service teachers’ willingness to implement DPA in their future classrooms. Thus, although many pre-service teachers learn about DPA during their education, in-service teachers and their classrooms also play an important role in supporting the development of DPA skills in future teachers.

Several factors were related to pre-service teachers’ perceptions of DPA value. The more pre-service teachers perceived physical activity benefitting their own mental health, and their students’ mental and physical health, the more they valued DPA. These findings are consistent with previous research indicating that teachers who recognized the benefits of physical activity for themselves and for their students were more likely to value classroom physical activity [60,61,62,82]. Promisingly, research has also shown that those who value classroom physical activity are also more likely to implement it. The current study also found that pre-service teachers who viewed physical activity and physical fitness as important parts of their teacher identity, also valued DPA more. This is consistent with prior work showing that in-service teachers who identified as physically active were more likely to recognize the benefits of classroom physical activity and implement it more often [83]. Education and practicum experience were also related to how pre-service teachers valued DPA; pre-service teachers who had opportunities to learn about DPA during their education and those who had opportunities to observe DPA during practicum valued DPA more. This is similar to previous research in which pre-service teachers who were enrolled in physical activity courses held higher views of classroom physical activity [84,85,86]. Furthermore, pre-service teachers who observed students engaging in and enjoying DPA also rated DPA more favorably. Although most pre-service teachers viewed DPA positively, there is clearly an added benefit of firsthand experience. Although these correlations were significant, they were only weak to moderate in strength, suggesting that while there is a relationship between factors, knowing one factor gives only a weak to moderate prediction of the value of the other. Factors unrelated to pre-service teachers’ perceptions of DPA value included previous physical education experience, perceptions of pre-service education preparing them to lead DPA, perceptions of physical activity benefiting their own physical and overall health, personal physical activity behavior, and identifying as “athletic.” These findings suggest that pre-service teachers do not necessarily require a personal relationship with physical activity to value its importance in the classroom.

Several factors were related to pre-service teachers’ confidence implementing DPA. Similar to previous work, pre-service teachers who were more physically active and who considered themselves fit or athletic rated themselves as more confident implementing DPA [63,64]. Pre-service teachers who are more familiar with physical activity may feel better equipped to lead activities [64]. Further, pre-service teachers who indicated having more positive physical education experiences were more confident with DPA implementation. This finding is supported by social learning theory, in that life experiences provide information that influence individuals’ thoughts and behaviors [52]. Pre-service teachers who had positive experiences in physical education may use this as a primary reference point for assessing personal physical activity competence later in life [64]. Past physical education experience is also important to help develop appropriate motor skills necessary to perform and enjoy physical activities [87,88], potentially contributing to greater DPA implementation confidence. Additionally, pre-service teachers who had more opportunities to learn about DPA, and perceived their education prepared them for implementing DPA, rated themselves as more confident implementing DPA. Other work has also similarly shown that pre-service teachers were more confident in their ability to implement DPA following a classroom physical activity course [86]. This result reinforces the importance of including DPA education in formal learning, as it is a mechanism for improving confidence. The current study also found a positive relationship between the perceived benefit of physical activity for students’ mental health and confidence in implementing DPA. Similar to findings presented previously, although these correlations were significant, they were only weak to moderate in strength, cautioning any predictive assertions between variables. Additionally, pre-service teachers’ perceptions of physical activity being beneficial for their own mental, physical, and overall health and their students’ physical health was unrelated to confidence implementing DPA. In recent years, both research and popular media has placed a heavy emphasis on the importance of mental health. Thus, pre-service teachers may be aware of student mental health and factors such as physical activity that promote it, and less aware of the importance of physical health and factors that support student physical wellbeing. Furthermore, while previous work has suggested that confidence improves with practicum experience [3,89], the current study found no relationship between practicum experience and confidence implementing DPA. Education itself was more important than practicum experience in fostering confidence implementing DPA. Given the lack of consistency in receiving hands-on training, pre-service teachers may have viewed their education as providing sufficient guidance to support DPA implementation.

Interestingly, there was no change in pre-service teachers’ perceptions of DPA from the beginning to the end of their education programs. This is promising given that over 97% of pre-service teachers finished the program recognizing the value of DPA. However, pre-service teachers’ confidence in implementing DPA declined from the beginning to the end of the program. Although this finding is not in line with previous work which found an increase in confidence with DPA following training [3,86,89], it does relate back to the idea that pre-service teachers may have had an inflated sense of confidence at the beginning of their education. After learning about what DPA involves through their education and many experiencing insufficient hands-on training, pre-service teachers may have recalibrated their confidence after recognizing the skillset required to translate DPA knowledge into practice. This again highlights the need for quality practicum experiences that allow pre-service teachers to become familiar with implementing DPA so that confidence and self-efficacy can remain meaningfully high.

## 5. Limitations

There are a few limitations to consider. First, an ideal response rate for survey-based research is 50% [90] with the current study yielding response rates between 31 and 35% across the two surveys. Several recruitment attempts were made throughout the 1.5-month recruitment periods. This recruitment window was necessary however to ensure that we were capturing pre-service perceptions as close as possible to the beginning and end of their education program. Second, the longitudinal sample size was smaller than anticipated, with fewer respondents from Year 1 responding to the Year 2 survey. Again, the limited recruitment window may have contributed to fewer respondents, but it was necessary to limit potential exposure to unaccounted external variables that may have influenced pre-service teacher perceptions beyond their education program.

## 6. Conclusions

Overall, the current study provides insight into key characteristics and experiences of pre-service teachers that are important for DPA fidelity. The findings point to several factors that may play a pivotal role in fostering effective implementation of DPA including providing pre-service teachers with greater opportunities to learn about DPA and observe DPA during school placements, and encouraging pre-service teachers to develop a positive intrapersonal relationship with physical activity. Although over 96% of pre-service teachers viewed physical activity as beneficial for their own and their students mental and physical health, given that only 23% of in-service teachers in Ontario are currently implementing DPA, translating those positive views to effective DPA implementation requires more pre-service level exposure and training.

## Figures and Tables

**Table 1:** Pre-service teachers’ perceptions of physical activity as it relates to their own and their students’ health.

		Frequency (%)	N	Frequency (%)	N
		Year 1		Year 2	
Survey items					
PA is beneficial for my own mental health	Agree Disagree	110 (100) 0 (0)	110	73 (96.1) 3 (3.9)	76
PA is beneficial for my own physical health	Agree Disagree	110 (100) 0(0)	109	74 (97.4) 2 (2.6)	76
Daily PA beneficial for my overall health	Agree Disagree	110 (100) 0(0)	109	73 (97.3) 2 (2.7)	75
Important for me as a teacher to be physically active	Agree Disagree	106 (96.4) 4 (3.6)	110	71 (93.4) 5 (6.6)	76
Important for me as a teacher to be physically fit	Agree Disagree	(100) (80.9) 21 (19.1)	107	48 (63.2) 28 (36.8)	76
Role of physical activity in students’ health					
PA is beneficial for my students’ mental health	Agree Disagree	109 (100) 0(0)	109	74 (97.4) 2 (2.6)	76
PA is beneficial for my students’ physical health	Agree Disagree	109 (100) 0(0)	109	74 (97.4) 2 (2.6)	76

**Table 2:** Pre-service teachers’ perceptions of physical activity in the school and classroom.

		Frequency (%)	N	Frequency (%)	N
		Year 1		Year 2	
Survey items					
DPA improves physical health of students	Agree Disagree	110 (100) 0 (0)	110	75 (97.4) 2 (2.6)	77
DPA improves cognition in students	Agree Disagree	110 (100) 0(0)	110	75 (97.4) 2 (2.6)	77
I believe students would demonstrate improved academic performance after participating in a bout of DPA	Agree Disagree	108 (99.1) 1 (0.9)	109	76 (98.7) 1 (1.3)	77
DPA is detrimental to cognitive functioning of students	Agree Disagree	27 (24.5) 83 (75.5)	110	16 (21.6) 58 (78.4)	74
Increase PA during school day will be detrimental to students’ academic success	Agree Disagree	8 (7.3) 101 (92.7)	109	5 (6.7) 70 (93.3)	75
Students should have opportunities for PA outside recess and physical education class	Agree Disagree	108 (99.1) 21 (0.9)	109	74 (97.4) 2 (2.6)	76
Teachers should play a role in PA programs at school	Agree Disagree	103 (94.5) 6 (5.5)	109	72 (94.7) 4 (5.3)	76
Teachers can make a difference helping children adopt lifelong PA habits	Agree Disagree	105 (96.3) 4 (3.7)	109	70 (92.1) 6 (7.9)	76
Teachers should provide DPA for students as part of school day	Agree Disagree	108 (99.1) 1 (0.9)	109	71 (94.7) 4 (5.3)	75

**Table 3:** Pre-service teachers’ experience with DPA implementation during their educational training.

		Frequency (%)	N
Survey items			
I had opportunities to observe DPA during practicum	Agree Disagree	49 (66.2) 25 (33.8)	74
Students were engaged in DPA *	Agree Disagree	45 (95.7) 2 (4.3)	47
Students enjoyed DPA *	Agree Disagree	45 (97.8) 1 (2.2)	46
I had opportunities to learn about DPA during pre-service education	Agree Disagree	54 (73) 20 (27)	74
My pre-service education prepared me for teaching DPA	Agree Disagree	51 (69.8) 21 (29.2)	72

*Note*. * Only participants who had practicum experiences with DPA answered these items.

**Table 4:** Key factors related to pre-service teachers’ perceptions of DPA value and confidence implementing DPA.

Key Factors Correlated	Perceived DPA Value r (*p*)	N	Confidence Implementing DPA r (*p*)	N
PA is beneficial for my own mental health	0.213 (*0.026)* *	109	0.073 (*0.452*)	108
PA is beneficial for my own physical health	0.134 (*0.168)*	109	0.142 (*0.144*)	107
Daily PA beneficial for my overall health	0.104 (*0.283)*	109	0.117 (*0.231*)	107
Important for me as a teacher to be physically active	0.308 (*0.001*) **	109	0.262 (*0.006*) **	108
Important for me as a teacher to be physically fit	0.267 (*0.006*) **	106	0.282 (*0.003*) **	106
PA is beneficial for my students’ mental health	0.393 (<*0.001*) **	109	0.253 (*0.009*) **	107
PA is beneficial for my students’ physical health	0.259 (*0.007*) **	109	0.118 (*0.224*)	107
Personal physical activity behavior (mins/week) (M = 264.1, SD = 259.3)	−0.066 (*0.502*)	106	0.191 (*0.05*) *	104
Physical activity identity	0.138 (*0.152*)	109	0.497 (<*0.001*) **	108
Previous physical education experience	0.064 (*0.513*)	106	0.453 (<*0.001*) **	106
I had opportunities to observe DPA during practicum	0.314 (*0.006*) **	74	0.055 (*0.647*)	71
Students were engaged in DPA	0.491 (<*0*.*001*) **	47	0.257 (*0.089*)	45
Students enjoyed DPA	0.301 (*0.042*) **	46	0.124 (*0.424*)	44
I had opportunities to learn about DPA during pre-service education	0.261 (*0.025*) *	74	0.289 (0.015) *	70
My pre-service education prepared me for teaching DPA	0.086 (*0.471*)	72	0.238 (*0.05*) *	68

*Note.* M = Mean, SD = Standard Deviation. * *p* < 0.05; ** *p* < 0.01.

## Data Availability

The data presented in this study are available on request from the corresponding author. The data are not publicly available due to ethical restrictions.
